# AI-assisted crop improvement: new design tools within established regulatory frameworks

**DOI:** 10.3389/fpls.2026.1882021

**Published:** 2026-09-14

**Authors:** Felicity Keiper, Mitscheli Sanches da Rocha, Delphine Sylvie Anne Beeckman, Ana Atanassova

**Affiliations:** 1 BASF Agricultural Solutions Australia Pty Ltd., Southbank, VIC, Australia; 2 BASF Agricultural Solutions US LLC, Research Triangle Park, NC, United States; 3 BASF Agricultural Solutions Belgium NV, Ghent, Belgium

**Keywords:** artificial intelligence, biotech crops, biotech regulation, crop improvement, genome editing, plant breeding, synthetic biology, AI

## Abstract

The integration of Artificial intelligence (AI) into crop improvement offers the potential to enhance the precision, efficiency, and speed of development of new varieties. Accelerated genetic gain is promised by an increasing repertoire of AI tools for analyzing large and complex genetic and phenotypic datasets to discover and elucidate traits and predict functional variants that can be realized with the use of, *inter alia*, genome editing tools. This power, in combination with parallel AI tool development for optimization of genome editing processes, has generated optimism for a new era of smart breeding. This Perspective examines the regulatory implications of AI-assisted crop improvement, using illustrative examples where deep learning models have been integrated for protein sequence and structure prediction, and the optimization or design of proteins for improved or novel functionalities. The examples represent either end of a spectrum of current and emerging genome editing applications: (i) editing of endogenous genes to enhance beneficial alleles and optimize functionality; and (ii) the design and engineering of new (“*de novo*”) protein domains for customized or new functionality. This range of outcomes can also be achieved without guidance from AI tools, albeit less efficiently, and their regulatory status is generally established. We consider relevant risks associated with these applications and contend that the regulatory status of the potential outcomes should not change, nor should they challenge the foundational applicability of existing regulatory frameworks and approaches for biotech crops. We also briefly discuss some current limitations of AI tools, and broader regulatory and governance considerations.

## 1 Introduction

Genome editing technologies are widely reported as having significant potential to transform crop breeding and trait development (Jiang et al., 2025; Chen et al., 2024b; Zhang et al., 2018). Since the first demonstrations of genome editing in plants (e.g. Lloyd et al., 2005; Shukla et al., 2009), plant scientists have embraced these tools for agriculture, with thousands of publications describing applications in plants (Hamdan et al., 2022; Wei et al., 2022). These applications are dominated by CRISPR-based approaches, demonstrating its versatility in plant breeding and for addressing existing breeding bottlenecks (e.g. increased crop yields, abiotic stress tolerances, resistances to pests and diseases, and nutritional quality) in a variety of fruit, vegetable, grain and oilseed crops (FAO, 2022; Adane and Alamnie, 2024; Chen et al., 2024a).

More recently, advances in Artificial Intelligence (AI) have elicited further excitement about a new era of “smart breeding” (Xu et al., 2022). In genome editing, developments in machine learning are supporting the optimization of editing parameters and procedures, thereby enhancing its efficiency, precision and the attainment of desired outcomes (Chen et al., 2024b). Active areas of AI model development include the identification of gene targets, optimizing the efficiency and precision of editing tools (e.g. prediction of on- and off-target edits, optimization of guide RNA designs, and optimization of CRISPR-Cas enzyme variants), predicting the phenotypic outcome of edits, and in the design of completely novel DNA or protein sequences (Abadi et al., 2017; Chen et al., 2024a; Dixit et al., 2024). These developments, in combination with recent advances in deep learning, provide the power to predict a wide range of functional variants which could then be tested and realized using genome editing tools (Wang et al., 2020).

Despite the rapid uptake and integration of genome editing technologies into crop improvement research and development (R&D) pipelines, few genome-edited crops have reached the market. The practical reality is that genome editing is technically complex and challenging in plants (Jiang et al., 2025; Chen et al., 2024a). In addition, the necessary global regulatory environment to enable commercial deployment of edited crops has evolved in a relatively slow and unharmonized manner (Fernández Ríos et al., 2025; Tachikawa and Matsuo, 2023; 2024). Consequently, commercial examples are limited to those managed within jurisdictions where regulatory status is clear, and the regulatory requirements of genetically modified organisms (GMOs) do not apply, e.g., High-GABA tomato in Japan (Ezura, 2022; Waltz, 2022) and mustard greens with reduced bitterness in the United States (Singleman, 2024). Additional examples are reported as having completed applicable regulatory procedures toward domestic commercial production, e.g. non-browning banana in the Philippines (non-GMO determination) (Jalaluddin et al., 2024; see review by Ahmar, 2026).

Generally, jurisdictions that have clarified regulatory status for applications of genome editing have either adapted pre-existing GMO regulatory frameworks or created *sui generis* administrative or legislative measures to manage products of “new breeding techniques” including genome editing (Jenkins et al., 2021; Turnbull et al., 2021). A global trend has emerged whereby process-based regulatory approaches developed for “older” recombinant-DNA technologies have evolved toward more risk-proportionate, product-focused oversight for “newer” technologies. For plants, this reflects a consensus that certain genetic changes (edits) could arise naturally (e.g. spontaneous mutation) or through conventional breeding (including induced mutation), and are equivalent to conventional/are “conventional-like” in terms of risk/safety considerations, and it would therefore be a disproportionate regulatory burden to apply GMO requirements (e.g. DEFRA, 2025; EC, 2023; EFSA, 2020; FDA, 2024; FSANZ, 2021; HC, 2024; OGTR, 2025). This consensus is supported by the long history of safe plant breeding practices and more than three decades of accumulated experience with the safe commercial use of GM crops (Keiper and Atanassova, 2022; Goberna et al., 2022). It is further informed by empirical evidence for risk being associated with the characteristics of the breeding outcome, and not the tools used in its development (Fernández Ríos et al., 2025). Conversely, edits that involve the introduction of “foreign” DNA sequences (refers to sequences originating from a non cross-compatible species) typically remain subject to case-by-case regulatory review as with GM plants (Keiper and Atanassova, 2022; Tachikawa and Matsuo, 2023).

In this Perspective, we reflect on the regulatory considerations arising from the integration of AI tools in crop improvement, specifically focusing on deep learning models (such as AlphaFold) for protein sequence and structure prediction and the optimization or design of proteins for improved or new functionalities. As a technology developer, this reflection is informed by more than three decades of experience with the regulation of GM crops throughout their development and commercialization. We contend that, despite perceptions around the novelty of AI tools and the potential outcomes when integrated into biotech applications, currently foreseeable outcomes do not inherently challenge established biotech regulatory frameworks, and the fundamental regulatory principles and considerations remain appropriate. However, the realization of the potential of these tools in crop improvement relies on continuing the trend prompted by genome editing regulations – toward a risk-proportionate and adaptive regulatory approach focused on the characteristics of the breeding outcome rather than the tools used in its development. We support this view by examining examples of current applications of AI-assisted crop improvement. We also consider current limitations linked to AI tools, and broader regulatory and governance issues.

## 2 AI and genome editing – from conventional-like edits to *de novo* protein design

The integration of AI tools in crop improvement can be broadly categorized into two operational modes: firstly, process optimization tools that streamline R&D, breeding and plant selection workflows; and secondly, design tools used in the development of new or improved traits. In this Perspective, we focus on the latter category, where machine learning assists the identification of genes and associated regulatory elements amenable to crop improvement. Active areas of investigation include, e.g.: (a) the prediction of molecular phenotypes, such as methylation status, histone modifications, and chromatin status (Gupta et al., 2025; Guo et al., 2025; Lv et al., 2026; Wang et al., 2021); (b) identifying cis-regulatory elements and predicting gene expression (Peleke et al., 2024); (c) modeling transcription sites and identifying editing target sites (Wang et al., 2020; Chen et al., 2024a); and (d) predicting the regulatory effects of genomic variants (Zhao et al., 2021). More recently, deep-learning tools such as AlphaFold have expanded the possibilities for protein modeling by providing accurate predictions of protein structures directly from amino acid sequences and improving the reliability of structure-based designs (Nussinov et al., 2022; Varadi et al., 2021).

To examine regulatory implications, we have selected illustrative examples from recently published literature that we consider to be on either end of the spectrum of current capabilities of AI-assisted crop improvement. At one end, protein modeling is used to pinpoint beneficial mutations to guide genome editing strategies, with outcomes akin to accelerated conventional breeding, i.e., identification of new allelic variation within the species’ gene pool. Two recent soybean (*Glycine max* L.) examples demonstrate the potential for AlphaFold-2 guided design. In Xia et al. (2024), a soybean pectin methylesterase inhibitor was edited to specifically target and inhibit a pectin methylesterase (PsPME1) secreted by a pathogen (*Phytophthora sojae*) that weakened plant cell walls. In prior biotechnological approaches, constitutive expression of the soybean pectin methylase inhibitor (GmPMI1) presented a trade-off between resistance and plant growth due to plant methylesterases also being inhibited. In this work, nine specific mutations were identified to design a modified form (GmPMI1R) to target only the pathogen’s enzyme, and its transient expression provided enhanced broad-spectrum resistance to oomycetes and fungal pathogens and normal plant growth (Xia et al., 2024). In the work of Wang et al. (2025), the functionality of a soybean sugar transporter (GmSWEET10a/b) was optimized by the introduction of deletions in low-oil alleles that block sugar transport, converting them into high-oil alleles, and mimicking the open-pore conformation of native high oil variants. Increased seed oil content was observed across multiple years and field trial sites without yield penalties (Wang et al., 2025). In both soybean examples, the type of edits described and resulting crops would be excluded from GMO regulatory oversight in many jurisdictions.

At the other end of the spectrum, deep-learning tools are increasingly enabling exploration of protein structures and functions beyond natural templates. Models including BindCraft, AlphaFold, RoseTTAFold, RFdiffusion, and ProteinMPNN have been applied to protein structure prediction, design, and sequence optimization, mostly for potential human therapeutic applications, e.g. newly designed (*de novo*) protein binders to accelerate screening cycles and improve success rates. In contrast, clearly demonstrated crop-focused applications remain limited (Iqbal et al., 2026). A potential future application of *de novo*–designed protein binders could involve an iterative workflow combining target selection, structural modeling, and computational design. In such a framework, structural information on plant- or pathogen-derived target proteins obtained experimentally or predicted using structure prediction tools (e.g., AlphaFold) could inform the *de novo* design of candidate binding proteins, with backbone generation achieved using generative design approaches (e.g., RFdiffusion) followed by sequence optimization (e.g. ProteinMPNN). The present key bottlenecks include the scarcity of plant-specific training data, the lack of evaluation metrics tailored to plant biology, and the need for scalable experimental validation frameworks capable of linking designed proteins to reproducible *in planta* phenotypes (Iqbal et al., 2026).

While the area is advancing, most tangible AI-assisted protein-design efforts in crop improvement currently focus on reengineering existing proteins rather than true *de novo* protein designs. In a recent study, a large family of plant receptor proteins (XII leucine-rich repeat receptor-like kinases) were mapped across 285 plant genomes to identify new candidate pattern recognition receptors, which are known to mediate resistance against pathogens and pests. Through testing hundreds of receptors, one from *Citrus maxima* (pomelo) recognized multiple bacterial species by targeting a conserved area (csp15) in cold shock proteins. Using AlphaFold protein modeling of receptor variants binding to csp15, modified versions of this receptor with broader recognition specificity were created with the capability of detecting pathogens that natural receptors missed. This work demonstrates a new strategy for enhanced disease resistance (Ngou et al., 2025), which could theoretically be realized via two routes: editing for targeted redesign of similar receptors in endogenous sequences – the outcomes of which would be considered conventional-like in many jurisdictions – or by introducing a newly designed receptor via transgenesis, resulting in a plant that would be within the scope of existing regulatory frameworks applicable to GMOs.

## 3 Regulatory considerations for AI-guided crop improvement

The continuum of AI-assisted outcomes highlighted above (section 3), ranging from “conventional-like” edits to enhance specific traits to engineered/reengineered proteins or domain combinations, indicate a broad range of potential uses in crop improvement. Established biotech regulatory frameworks can accommodate this spectrum: conventional-like edits are often exempt from GMO regulation, while transgenic outcomes, including *de novo* designed proteins, remain within the scope of GMO regulatory provisions. The integration of AI tools as presently and foreseeably applied should not challenge this regulatory status, as they do not inherently create novel regulatory categories or risk considerations.

The use of new tools and the potential resulting off-targets have been a concern of those advocating for continued process-based regulation, with this view based in perceived inherent risks of the tools themselves (e.g. Eckerstorfer et al., 2019). However, the potential role of AI predictive modeling in early identification of potential risks and risk mitigation strategies has been identified (e.g. Lou et al., 2026), and AI-assisted optimization of editing processes and outcomes could reduce unintended outcomes, such as off-target edits, especially in complex crop genomes. Thus, from a regulatory perspective, AI can be viewed as a supportive tool that, when accompanied by the appropriate transparency, model validation, and reproducibility (see section 4), may further inform problem formulation approaches to risk assessment – an approach that is recognized internationally as best practice (see, e.g. OECD, 2023a; SCBD, 2025).

For applications involving insertion of foreign DNA, including *de novo* synthesized DNA (assembled without using a physical natural DNA template), established regulatory practice for GMOs is equipped to assess novel genes/proteins regardless of their source. Risk/safety assessment addresses the risk profile of the protein (e.g. toxicity, allergenicity) and on the resulting GM plant or products thereof according to established *principles* (e.g. case-by-case approach, familiarity, weight-of-evidence, problem formulation) and *parameters* (e.g. molecular and protein characterization, analyses of nutritional composition, ecotoxicology, and agronomic performance; see Codex, 2003b; OECD, 2023a; 2023b; 2025a). Per current regulatory best practice, the nature and extent of evidence required should be guided by a problem-formulation approach and testing of risk hypotheses. This practice is reviewed in the literature (e.g. Koch et al., 2025; Turnbull et al., 2021) and reflected in international law and policy developments (e.g. Cartagena Protocol; SCBD, 2000), standards (e.g. ISO, 2006), guidance and best practices (e.g. Codex, 2003a; 2003b; OECD, 2023a), aimed at supporting the safe and responsible development and adoption of innovation.

Over the past decade, relevant policy discussions on “synthetic biology” (or similar, e.g. “engineering biology”, “emerging biotechnologies”) have examined the potential challenges for comparator-based risk assessment practice in cases where a GMO differs substantially from its non-modified counterpart (such as a parental or host organism, often a near isogenic plant line for GM crop risk assessment, see Kleter et al., 2023), or where traits are considered novel and unfamiliar, and lack a history of safe use (e.g. Scientific Committees, 2015). In such cases – which are possible with and without the use of AI – additional hypothesis-driven evidence may be needed to inform regulatory assessment on a case-by-case basis. The policy discourse highlights the need for greater understanding of biological functions, improved modeling, and tools for predicting emergent properties (e.g. Scientific Committees, 2015). All of these identified areas are now benefitting from developments in AI tools, however their limitations and need for continual improvement must also be recognized, e.g. the need for comprehensive training libraries to drive model learning, rigorous validation of predicted structures, and bias mitigation strategies (Koh et al., 2025; Li et al., 2025). While these technical issues are not insurmountable, they demonstrate that the capabilities of AI are not yet fully realized, and areas remain for future innovation (Koh et al., 2025).

## 4 Discussion

In this Perspective we contend that the integration of AI as a supportive tool in crop improvement does not inherently introduce novel risk considerations, and the outcomes (i.e. edited conventional-like or GM plant) can be assessed according to established science-based risk assessment approaches where applicable. In the examples presented in section 2, the regulatory status of the products should not be challenged, particularly for regulatory frameworks trending toward product and outcome-based assessments. These examples demonstrate that the integration of AI tools expands the genetic variability available for crop improvement and should be viewed as part of the continuum and refinement of technological development and innovation and not a disruption to regulatory practice.

Whilst we contend that the safety of the potential end products in these examples can be adequately handled by existing regulatory frameworks and assessment paradigm (where applicable), we acknowledge that cross-cutting regulatory and governance challenges arise with the use of AI. Apart from technical limitations mentioned above (section 3), these include AI validation, transparency and explainability/interpretability, “dual-use” security concerns, maintaining human oversight (“human in-the-loop”) and judgement, and public acceptance and ethical considerations. Although these are not the focus of this Perspective, we note that AI explainability/interpretability is mentioned as a limitation relevant to risk/safety assessment in current literature (e.g. Groff-Vindman et al., 2025; see also OECD, 2025b; WHO, 2021). This is attributable to the “black box” operation of some AI tools, whereby predictions are generated without transparent reasoning and may include flawed outputs. The need for greater transparency in the computational reasoning processes in future versions of AI tools has been highlighted as necessary for users to track and troubleshoot these limitations (Koh et al., 2025). It is also important to note that for crops, this is only an early point in a lengthy R&D process that involves extensive phenotypic characterization and testing of trait performance with selection over multiple generations, as well as molecular and protein characterization studies required to support regulatory assessment (where applicable) – editing outcomes that are inconsistent with predictions would be readily identified and eliminated from further development.

Another cross-cutting area is the use of AI to improve efficiency in regulatory processes and practices more broadly, including regulatory affairs (e.g. dossier preparation, filing, review), compliance, and decision-making (e.g. Ajmal et al., 2025; also Hartung, 2023; Luechtefeld and Hartung, 2025). As AI technology and capabilities evolve and become commonplace in R&D pipelines, AI data may be provided to support risk/safety assessment. Therefore, regulatory agencies will need to strengthen their AI literacy and adapt their oversight processes, including safeguards to avoid overly positive feedback loops or echo chambers in case the same AI tool is used for both dossier preparation and reviewing of applications (Hosseini et al., 2025). This has been recognized by agencies that regulate drugs (United States Food and Drug Administration (FDA), European Medicines Agency, and the Medicines and Healthcare products Regulatory Agency of the United Kingdom) as well as the food products of GM crops (FDA, and the European Food Safety Authority (EFSA)) (EFSA, 2025). In a pioneering step, in January 2025 the FDA issued a draft guidance for stakeholders proposing a risk-based credibility assessment framework for evaluating AI models and establishing that they are fit for purpose in regard to data that supports product safety and effectiveness (FDA, 2025a). In parallel, the FDA undertook an agency-wide rollout of generative AI tools for scientific reviewers (FDA, 2025b).

In conclusion, it is evident that the integration of AI tools in crop improvement will expand biotech capabilities and strategies for crop improvement, and we contend that the outcomes can be managed according to established biotech regulatory practice. However, we also view the cross-cutting considerations relevant to responsible technology development and the realization of potential benefits. With increasing integration of AI tools into both R&D *and* applicable regulatory processes, there is need to ensure transparency and traceability regarding the context in which AI was used and in the validation of results, and human oversight in design processes and decision-making.

## Data Availability

The original contributions presented in the study are included in the article/supplementary material. Further inquiries can be directed to the corresponding author.
